# Supplementary material for: Dysregulation of FURIN and Other Proprotein Convertase Genes in the Progression from HPV Infection to Cancer
Source: Int J Mol Sci. 2025 Jan 8;26(2):461. doi: 10.3390/ijms26020461 (PMC11764867; doi:10.3390/ijms26020461)
Supplement: Supplementary file 1 [file ijms-26-00461-s001.zip › ijms-3388416-supplementary.pdf]

International Journal of
Molecular Sciences

MDPI

Supplementary Materials

Dysregulation of FURIN and Other Proprotein Convertase Genes in the Progression from HPV Infection to Cancer

Gonzalo Izaguirre [1,*], Natalia Zirou [1] and Craig Meyers [2]

[1] Department of Periodontics, College of Dentistry, University of Illinois Chicago, Chicago, IL 60612, USA
[2] Departments of Microbiology and Immunology, College of Medicine, Penn State University, Hershey, PA 17033, USA
* Correspondence: goniza@uic.edu

2. Results

2.4. Bioinformatic analysis of large transcriptomic datasets- normal cells

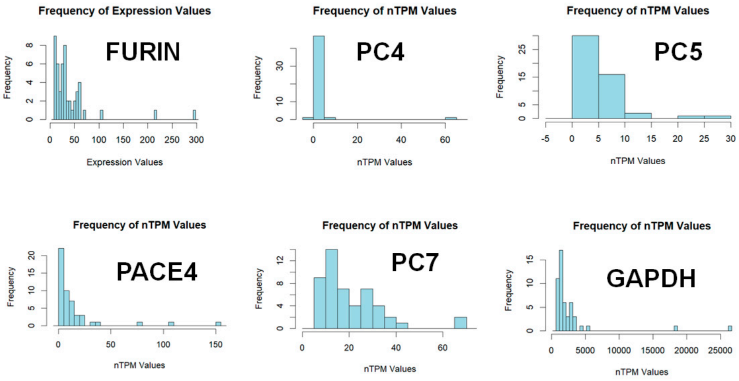

Figure S1. *Frequency distribution of PC gene expression counts in normal tissues.* Publicly available data sets (HPA, GTEx) containing the transcriptome expression counts of 50 healthy tissues were analyzed.

Table S1. **PC gene transcription expression counts from normal human tissues**. Frequency distribution of PC gene expression counts (nTPM) in normal tissues. Publicly available data sets (HPA, GTEx) containing the transcriptome analysis of 50 healthy tissues were accessed and analyzed. The plotted data is shown in Figure S1.

	Modal bin of RNA expression (nTPM)	Modal Frequency out of 50 tissues	Lower expression end	Upper expression end
FURIN	9 – 13	9	9	300
PC4	0 – 5	47	0	64
PC5	0 – 5	30	0	25
PACE4	0 – 5	22	0	160
PC7	10 – 15	14	5	67
GAPDH	1,100 – 1,600	17	630	27,000

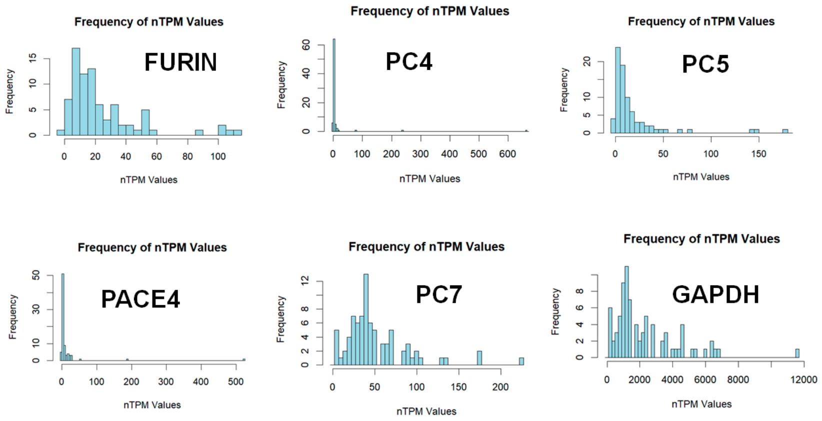

Figure S2. *Frequency distribution of PC gene expression counts in normal single cells.* A publicly available data set (HPA) containing the transcriptome expression counts of single cell suspensions from 81 healthy tissues was analyzed.

Table S2. **PC gene transcription expression counts from human normal single cells**. Frequency distribution of PC gene expression counts (nTPM) in normal tissue single cells. A publicly available data set (HPA) containing the transcriptome analysis of single cell suspensions from 81 healthy tissues was accessed and analyzed. The plotted data is shown in Figure S2. Lower and higher end expression values are expressed as the mean and standard deviation.

	Modal bin of RNA expression (nTPM)	Modal Frequency out of 82 cell lines	Lower expression end. Average of the bottom 2 - 5 cell lines	Upper expression end. Average of the top 3 - 5 cell lines
**FURIN**	5 – 10	17	0.27 ± 0.20	106 ± 5
**PC4**	0 – 5	64	0.0 ± 0.0	452 ± 212
**PC5**	0 – 5	24	0.0 ± 0.0	156 ± 17
**PACE4**	0 – 5	51	0.0 ± 0.0	355 ± 168
**PC7**	37 – 42	13	5.6 ± 1.7	189 ± 24
**GAPDH**	1080 -1280	11	106 ± 22	7,810 ± 2,147

2.5. *PC gene expression in cancer samples and cell lines*

Table S3. **Cancer types**. The table lists the 21 different cancer types included in the 7932 tissue samples studied.

Cancer type	Abbreviation	Number of samples
Blader carcinoma	BLCA	407
Breast carcinoma	BRCA	1074
Cervical Squamous Cell Carcinoma and Endocervical Adenocarcinoma	CESC	290
Colon adenocarcinoma	COAD	437
Glioblastoma	GBM	152
Head and neck squamous cell carcinoma	HNSC	498
Kidney Chromophobe renal cell carcinoma	KICH	63
Kidney renal cell carcinoma	KIRC	526
Kidney renal papillary cell carcinoma	KIRP	285
Liver hepatocellular carcinoma	LIHC	364
Lung adenocarcinoma	LUAD	499
Lung squamous cell carcinoma	LUSC	493
Ovarian cancer	OV	462
Pancreatic adenocarcinoma	PAAD	175
Prostate adenocarcinoma	PRAD	493
Rectal adenocarcinoma	READ	158
Skin cutaneous melanoma	SKCM	101
Stomach adenocarcinoma	STAD	353
Testicular germ cell tumors	TGCT	133
Thyroid carcinoma	THCA	500
Uterine corpus endometrial carcinoma	UCEC	540

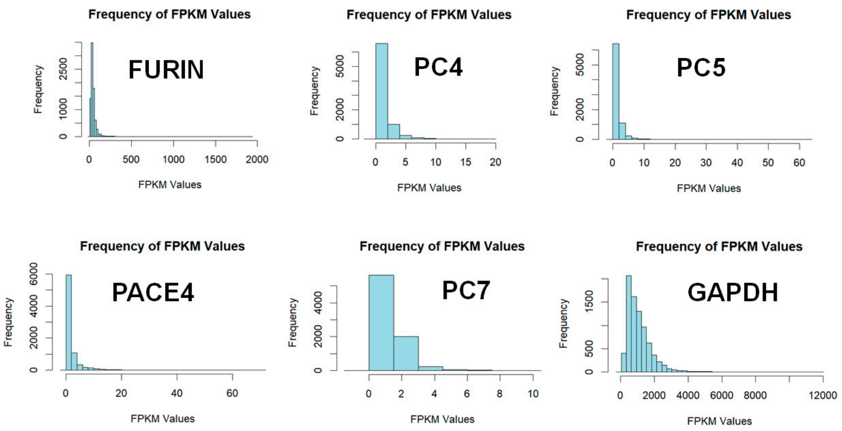

Figure S3. *Frequency distribution of PC gene expression counts in cancer tissue samples.* A publicly available data set (TCGA) containing the transcriptome expression counts of 7932 tissue samples belonging to 21 different cancer types was analyzed.

Table S4. **PC gene transcription expression counts from human cancer tissue samples**. Frequency distribution of PC gene expression counts (FPKM) in cancer tissue samples. A publicly available data set (TCGA) containing the transcriptome analysis of 7932 samples belonging to 21 different cancer types was accessed and analyzed. The plotted data is shown in Figure S3. Lower and higher end expression values are expressed as the mean and standard deviation.

	Modal bin of RNA expression (FPKM)	Modal Frequency out of 7932 samples	Lower expression end. Average of the bottom 15 samples	Upper expression end. Average of the top 15 samples
FURIN	21 – 41	3486	3.07 ± 0.20	821 ± 112
PC4	0 – 2	6569	0.0164 ± 0.0019	14.4 ± 0.558
PC5	0 – 2	6409	0.0184 ± 0.00156	23.4 ± 2.91
PACE4	0 – 2	5939	0.0127 ± 0.0010	43.8 ± 2.60
PC7	0 – 1.5	5626	0.0762 ± 0.00424	7.17 ± 0.146
GAPDH	337 – 637	2066	108 ± 6.66	6307 ± 452

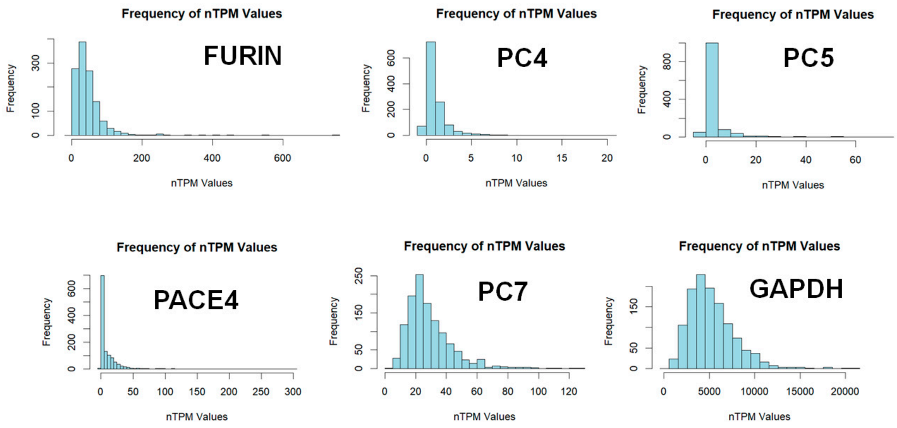

Figure S4. *Frequency distribution of PC gene expression counts in cancer cell lines.* A publicly available data set (HPA) containing the transcriptome expression counts of 1206 cancer cell lines was analyzed.

Table S5. **PC gene transcription expression counts from human cancer cell lines.** Frequency distribution of PC gene expression counts (nTPM) in cancer cell lines. A publicly available data set (HPA) containing the transcriptome analysis of 1206 cancer cell lines was accessed and analyzed. The plotted data is shown in Figure S4. Lower and higher end expression values are expressed as the mean and standard deviation.

	Modal bin of RNA expression (nTPM)	Modal Frequency out of 1206 cell lines	Lower expression end. Average of the bottom 15 cell lines	Upper expression end. Average of the top 15 cell lines
FURIN	20 – 40	387	1.8 ± 0.2	346 ± 37
PC4	0 – 1	725	0 ± 0	8.2 ± 0.8
PC5	0 – 5	997	0 ± 0	47.1 ± 2.6
PACE4	0 – 5	696	0.05 ± 0.01	139 ± 17
PC7	20 – 25	253	7.7 ± 0.3	97 ± 4
GAPDH	3550 – 4550	228	1,084 ± 70	15,870 ± 708

2.6. Cancer leads to dysregulation of PC gene expression

Table S6. **Mean PC gene expression values across normal and cancerous tissues and cells.** Mean gene expression counts in Figure 5 were standardized to the expression values of the GAPDH gene as described in the Results section. Percentage out of the total mean PC gene expression is shown in parenthesis.

	Normal Tissues	Normal Single Cells	Cancer Samples	Cancer Cell Lines
FURIN	154 ± 185 (48.6)	108 ± 110 (19.8)	413 ± 493 (88.2)	88.0 ± 90.5 (52.1)
PC4	56.0 ± 186 (17.7)	64.9 ± 355 (11.9)	18.8 ± 21.0 (3.9)	19.2 ± 23.7 (11.4)
PC5	10.1 ± 9.7 (3.2)	63.6 ± 110 (11.7)	11.9 ± 16.6 (2.5)	2.0 ± 4.9 (1.2)
PACE4	19.7 ± 36.2 (6.2)	80.4 ± 338 (14.7)	12.5 ± 23.6 (2.7)	5.0 ± 10.0 (3.0)
PC7	77.7 ± 52.2 (24.5)	228 ± 172 (41.8)	12.1 ± 7.7 (2.6)	55.0 ± 28.6 (32.5)
GAPDH	2663 ± 4228	2250 ± 1986	1078 ± 711	5274 ± 2566

Table S7. **Modal PC gene expression values across normal and cancerous tissues and cells.** Comparison of data in Tables S1, S2, S4, and S5. Modal gene expression counts in each table were standardized to the expression values of the GAPDH gene as described in the Results section. Percentage out of the total modal PC gene expression is shown in parenthesis.

	Normal Tissues	Normal Single Cells	Cancer Samples	Cancer Cell Lines
FURIN	48.3 (49.8)	132 (5.6)	13, 700 (99.4)	258 (80.9)
PC4	11.9 (12.3)	704 (30.0)	14.5 (0.10)	0.2 (0.063)
PC5	3.0 (3.1)	91.2 (3.9)	23.1 (0.17)	7.5 (2.35)
PACE4	13.9 (14.3)	441 (18.8)	39.9 (0.29)	15.4 (4.83)
PC7	19.9 (20.2)	975 (41.6)	4.7 (0.03)	38.2 (12.0)
GAPDH	6.34 x 10e+6	6.26 x 10e+5	4.11 x 10e+5	6.49 x 10e+6

2.7. FURIN gene expression determines different cancer phenotypes

Table S8. **Low and high FURIN gene expressing cancer cell lines**.

Cell line	Disease	Cancer type	Primary / Metastasis	mRNA expression (nTPM)				
				FURIN	PC4	PC5	PACE4	PC7
NCI-H2126	Lung cancer	NSCLC Adenocarcinoma	Metastasis/lymp hoid tissue	752	4.7	1.0	0.5	26.3
HuT 102	Lymphoma	T-Cell	------	549	0.4	0.5	0.5	39.4
Karpas-299	Lymphoma	T-Cell, Non-Hodgkin	------	440	2.6	0.2	0.9	23
Huh-7	Liver cancer	Hepatocellular carcinoma	Primary	417	0.6	8.8	113	16
Fu97	Gastric cancer	Adenocarcinoma	Primary	380	0.3	0.1	167	12.5
HCC2302	Lung cancer	---------	-----------	339	0.9	0.2	6.6	43.1
Cell line	Disease	Cancer type	Primary / Metastasis	mRNA expression (nTPM)				
				FURIN	PC4	PC5	PACE4	PC7
GA-10	Lymphoma	B-Cell, Non-Hodgkins	--------	0.6	0.1	0.2	0.8	38.0
NU-DUL-1	Lymphoma	Large B-Cell	--------	0.7	0.6	0.2	0.4	27.5
Ramos	Lymphoma	B-Cell, Non-Hodgkins	--------	0.7	0.1	0.1	0.4	27.3
BL-70	Lymphoma	B-Cell, Non-Hodgkins	--------	0.8	0.1	0.3	0.6	43.8
Daudi	Lymphoma	-------	-------	1.1	0.8	0.3	0.4	23.0
MC116	Lymphoma	B-Cell, Non-Hodgkins	--------	1.5	1.0	0.2	0.4	27.3

Table S9. **Low and high PC4 gene expressing cancer cell lines**.

Cell line	Disease	Cancer type	Primary / Metastasis	mRNA expression (nTPM)				
				FURIN	PC4	PC5	PACE4	PC7
SBC-5	Lung cancer	SCLC	Metastasis/pleur a	63.0	19.1	0.2	16.9	18.1
HPB-ALL	Leukemia	T-Cell, ALL	Primary	17.1	11.0	5.0	0.6	39.5
SNU-1079	Bile duct cancer	Cholangiocarcino ma	--------	17.9	8.7	0.5	11.3	12.4
HEC-108	Uterine cancer	Endometrial adenocarcinoma	Primary	26.2	8.6	4.4	0.7	18.4
JURKAT	Leukemia	--------	Primary	16.1	7.9	1.7	4.4	25.2
U-266/70	Myeloma	--------	--------	14.3	7.8	0.1	0.5	13.8
Cell line	Disease	Cancer type	Primary / Metastasis	mRNA expression (nTPM)				
				FURIN	PC4	PC5	PACE4	PC7
A-375	Skin cancer	Melanoma	Primary	46.7	0.0	1.3	0.9	29.9
CAL-85-1	Breast cancer	Carcinoma	Primary	179	0.0	0.1	32.7	31.2
COLO 794	Skin cancer	Melanoma	Primary	88.2	0.0	5.3	3.2	28.2
EB3 [........]	Lymphoma	B-Cell, Non-Hogdkins	------	3.0	0.0	0.1	0.3	42.2
ECC12	Gastric cancer	Small cell carcinoma	-------	29.1	0.0	0.3	0.4	27.9
FTC-238	Thyroid cancer	Carcinoma, follicular	Metastasis/Lung	46.7	0.0	0.1	0.3	25.2

Table S10. **Low and high PC5 gene expressing cancer cell lines**.

Cell line	Disease	Cancer type	Primary / Metastasis	mRNA expression (nTPM)				
				FURIN	PC4	PC5	PACE4	PC7
OVTOKO	Ovarian cancer	Adenocarcinoma	Metastasis/spleen	30.6	1.2	70.8	0.3	14.9
OVISE	Ovarian cancer	Adenocarcinoma	Matastasis/bone	27.0	3.6	59.0	1.9	10.8
A-498	Kidney cancer	Renal cell carcinoma	Primary	21.6	1.2	54.8	13.0	25.7
NUGC-4	Gastric cancer	Adenocarcinoma	Metastasis/lymph node	35.9	0.3	53.1	9.4	62.8
ME-180	Cervical cancer	Squamous cell carcinoma	Metastasis/abdomen	11.4	1.2	50.6	32.6	42.9
Caki-2	Kidney cancer	Renal carcinoma	Primary	28.8	1.3	50.2	4.4	11.4
Cell line	Disease	Cancer type	Primary / Metastasis	mRNA expression (nTPM)				
				FURIN	PC4	PC5	PACE4	PC7
888-mel	Skin cancer	Melanoma	Metastasis	41.1	0.0	0.0	16.8	23.3
BT-549	Breast cance	Breast ductal carcinoma	Primary	11.3	0.2	0.0	0.2	22.8
CAL-12T	Lung cancer	NSCLC	Primary	142	0.6	0.0	1.6	23.9
Evsa-T	Breast cancer	Carcinoma	Metastasis/ascites	41.0	0.3	0.0	1.1	25.6
EFM-192A	Breast cancer	Adenocarcinoma	Metastasis/pleura	23.3	0.5	0.0	5.6	23.9
HCC1569	Breast cancer	Carcinoma	Primary	65.5	0.1	0.0	37.4	32.5

Table S11. **Low and high PACE4 gene expressing cancer cell lines.**

Cell line	Disease	Cancer type	Primary / Metastasis	mRNA expression (nTPM)				
				FURIN	PC4	PC5	PACE4	PC7
Hs 255.T	Non-cancerous	Colorectal	---------	81.1	0.4	0.7	302	60.6
SK-MEL-1	Skin cancer	Melanoma	Metastasis/lymphoid tissue	36.9	0.6	0.1	261	32.0
Hs 675.T	Non-cancerous	Colorectal	-----------	91.8	0.5	8.0	185	39.3
Fu97	Gastric cancer	Adenocarcinoma	Primary	380	0.3	0.1	167	12.5
TE 441.T	Sarcoma	Rhabdomyosarcoma	--------	46.5	2.0	3.2	159	44.5
DU4475	Breast cancer	Carcinoma	Metastasis/skin	21.0	0.8	0.1	129	21.2
Cell line	Disease	Cancer type	Primary / Metastasis	mRNA expression (nTPM)				
				FURIN	PC4	PC5	PACE4	PC7
DOV13	Ovarian cancer	Adenocarcinoma	Primary	81.9	0.8	0.3	0.0	19.7
HEY	Ovarian cancer	Adenocarcinoma	-------	26.8	0.0	2.1	0.0	33.1
OVCA420	Ovarian cancer	Adenocarcinoma	Primary	36.7	0.0	22.7	0.0	24.0
NCE-G 118	Brain cancer	-------	Primary	35.5	0.3	0.7	0.0	28.4
Calu-1	Lung cancer	NSCLC, SCC	Metastasis/pleura	26.1	0.4	0.1	0.1	36.8
A4/Fukuda	Lymphoma	DLBCL	Primary	14.5	0.9	0.3	0.1	33.8

Table S12. **Low and high PC7 gene expressing cancer cell lines**.

Cell line	Disease	Cancer type	Primary / Metastasis	mRNA expression (nTPM)				
				FURIN	PC4	PC5	PACE4	PC7
HCC1263	Colorectal cancer	------	------	97.2	0.8	2.5	53.6	129
HCC1588	Lung cancer	NSCLC, SCC	Primary	54.5	0.8	2.0	56.0	124
G-292	Bone cancer	Osteosarcoma	Primary	40.2	0.2	0.9	8.4	114
CL-40	Colorectal cancer	Adenocarcinoma	Primary	27.5	0.2	0.3	73.1	109
HT	Lymphoma	B-Cell, Non-Hodgkins	------	17.4	0.9	0.1	0.3	97.6
SNU-61	Colorectal cancer	Adenocarcinoma	Metastasis/liver	18.5	0.4	3.0	41.4	95.4

Cell line	Disease	Cancer type	Primary / Metastasis	mRNA expression (nTPM)				
				FURIN	PC4	PC5	PACE4	PC7
HuCC-T1	Bile duct cancer	Cholangiocarcinoma	Metastasis/ascites	1.8	0.0	0.1	4.5	5.0
EC-GI-10	Esophageal cancer	SCC	Metastasis/lymph node	20.1	3.4	8.1	6.9	6.0
Mel 928	Skin cancer	Melanoma	-------	68.5	0.0	0.0	4.4	6.3
TE-4	Esophageal cancer	SCC	Metastasis/lymph node	102	0.4	3.2	0.4	6.6
U-343MGa	Brain cancer	Glioblastoma	Primary	61.0	3.4	1.9	0.7	6.9
MDA-MB-330	Breast cancer	Carcinoma	Metastasis/pleura	41.8	0.5	0.0	4.8	7.1

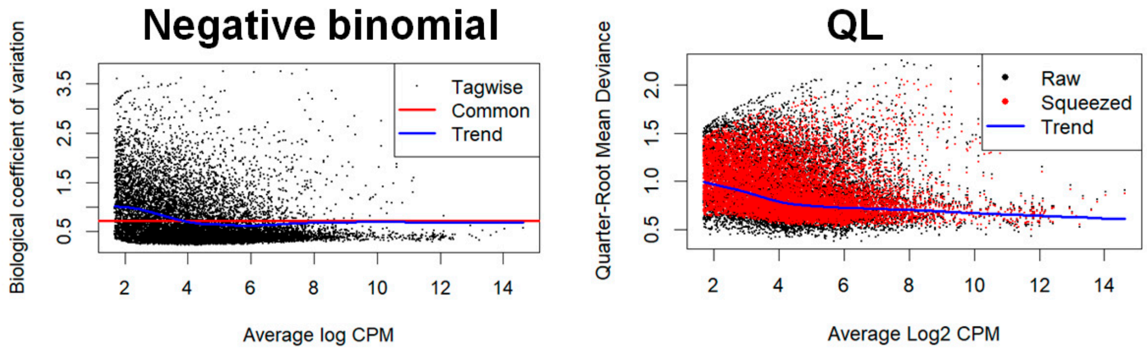

Figure S5. *Dispersion plots from the RNA-seq differential gene expression analysis of the high and low FURIN expressing cancer cell lines*. *Left Panel* - Mean-variance plot showing the dispersion across gene expression levels according to the Negative Binomial model. *Right Panel* - Mean variance plot showing the dispersion associated to the Quasi-likelihood model framework.

2.8. HPV infection related genes are differentially expressed between high and low FURIN expressing cancer cells

Table S13. **Gene ontology categories and subcategories.**

ID	Category	Subcategory	Description	GeneRatio	BgRatio	RichFactor	FoldEnrichment	zScore	pvalue	p.adjust	qvalue
hsa05205	**Human Diseases**	**Cancer: overview**	**Proteoglycans in cancer**	56/605	204/8850	0.2745098	4.015557	11.803326	2.82 e-20	8.28 e-18	4.55 e-18
hsa04510	Cellular Processes	Cellular community - eukaryotes	Focal adhesion	53/605	203/8850	0.2610837	3.819159	11.006882	3.55 e-18	5.20 e-16	2.85 e-16
hsa04151	Environmental Information Processing	Signal transduction	PI3K-Akt signaling pathway	70/605	362/8850	0.1933702	2.828638	9.622962	5.09 e-16	4.97 e-14	2.73 e-14
hsa04015	Environmental Information Processing	Signal transduction	Rap1 signaling pathway	50/605	212/8850	0.2358491	3.450023	9.780499	3.00 e-15	2.19 e-13	1.20 e-13
hsa04010	Environmental Information Processing	Signal transduction	MAPK signaling pathway	61/605	300/8850	0.2033333	2.974380	9.424055	4.06 e-15	2.38 e-13	1.30 e-13
hsa05165	**Human Diseases**	**Infectious disease: viral**	**Human papillomavirus infection**	64/605	333/8850	0.1921922	2.811406	9.126918	1.38 e-14	6.78 e-13	3.73 e-13

Table S14. **HPV infection associated genes.**

ENTREZID	SYMBOL		ENTREZID	SYMBOL
3675	ITGA3		2335	FN1
5291	PIK3CB		6772	STAT1
7132	TNFRSF1A		6696	SPP1
5590	PRKCZ		4853	NOTCH2
5526	PPP2R5B		8638	OASL
5566	PRKACA		3915	LAMC1
3993	LLGL2		7057	THBS1
5743	PTGS2		3685	ITGAV
5663	PSEN1		1950	EGF
2932	GSK3B		1280	COL2A1
7481	WNT11		207	AKT1
5829	PXN		1956	EGFR
3912	LAMB1		6934	TCF7L2
3655	ITGA6		3688	ITGB1
182	JAG1		83439	TCF7L1
8717	TRADD		89780	WNT3A

5595	MAPK3		8324	FZD7
5516	PPP2CB		90993	CREB3L1
5296	PIK3R2		1857	DVL3
3955	LFNG		3678	ITGA5
10488	CREB3		23462	HEY1
1855	DVL1		8323	FZD6
1277	COL1A1		1499	CTNNB1
9368	NHERF1		8313	AXIN2
7448	VTN		5986	RFNG
10312	TCIRG1		3913	LAMB2
11211	FZD10		8322	FZD4
7422	VEGFA		10134	BCAP31
1452	CSNK1A1		527	ATP6V0C
5159	PDGFRB		7477	WNT7B
3280	HES1		5649	RELN
3694	ITGB6		3690	ITGB3

4. Materials and Methods

4.3 Bioinformatics analysis

4.3.1 Code lines

4.3.1.1 Gene IDs

	ensembl_gene_id	hgnc_symbol
Furin	ENSG00000140564	FURIN
PC4	ENSG00000115257	PCSK4
PC5	ENSG00000099139	PCSK5
PACE4	ENSG00000140479	PCSK6
PC7	ENSG00000160613	PCSK7
GAPDH	ENSG00000111640	GAPDH

4.3.1.2 Expression count modal bins, modal frequencies, and lowest and highest range values

```
> furin_data <- subset(celline_nTPM_data, Gene == "ENSG00000140564")
> dim(furin_data)
> head (furin_data)
> breaks_seq <- seq(floor(min(furin_data$nTPM, na.rm = TRUE)) - 10.0, ceil-
  ing(max(furin_data$nTPM, na.rm = TRUE)) + 10.0, by = 10.0)
> hist(furin_data$nTPM, breaks = breaks_seq, main = "Frequency of nTPM Values", xlab
  = "nTPM Values", ylab = "Frequency", col = "lightblue", border = "black")
> binned_values <- cut(furin_data$nTPM, breaks = breaks_seq, include.lowest = TRUE)
> binned_value_frequencies <- table(binned_values)
> modal_bin <- names(binned_value_frequencies)[which.max(binned_value_frequen-
  cies)]
> modal_bin_frequency <- max(binned_value_frequencies)
> modal_bin
> modal_bin_frequency
> furin_data <- subset(celline_nTPM_data, Gene == "ENSG00000165704")
> top_15_rows <- furin_data[order(furin_data$nTPM, decreasing = TRUE), ]
> top_15_rows <- head(top_15_rows, 15)
> print(top_15_rows)
> expression_values <- top_15_rows$nTPM
> mean_expression <- mean(expression_values)
> standard_error <- sd(expression_values) / sqrt(length(expression_values))
> mean_expression
> standard_error
> bottom_15_rows <- head(furin_data[order(furin_data$nTPM), ], 15)
> print(bottom_15_rows)
> expression_values <- bottom_15_rows$nTPM
> mean_expression <- mean(expression_values)
> standard_error <- sd(expression_values) / sqrt(length(expression_values))
> mean_expression
> standard_error
```

4.3.1.2 Gene expression analysis

```
> furin_data <- read.delim("C://Furin_analysis/furin_wide_data_1.txt", row.names = 1)
> dim(furin_data)
> colnames (furin_data)
> new_column_names <- c("F1", "F2", "F3", "F4", "F5", "F6", "f1", "f2", "f3", "f4", "f5", "f6")
> colnames(furin_data) <- new_column_names
> ensg_ids <- furin_data$ENSG
> mart <- useMart("ensembl", dataset = "hsapiens_gene_ensembl")
> mapping <- getBM( attributes = c("ensembl_gene_id", "hgnc_symbol"), mart = mart )
> furin_data$ensembl_gene_id <- rownames(furin_data)
```

```
> merged_data <- merge(furin_data, mapping, by = "ensembl_gene_id")
> dim (merged_data)
> merged_data <- merged_data[!duplicated(merged_data$hgnc_symbol), ]
> dim(merged_data)
> rownames(merged_data) <- merged_data$hgnc_symbol
> merged_data <- merged_data[, !(colnames(merged_data) %in% c("ensembl_gene_id",
  "hgnc_symbol"))]
> dim(merged_data)
> filter_merged_data <- merged_data[rowSums(merged_data[, 1:12]) >= 20, ]
> dim (filter_merged_data)
> furin_merged_data <- filter_merged_data
> y <- DGEList(counts = furin_merged_data[, 1:12], genes =
  rownames(furin_merged_data))
> dim (y)
> head (y)
# Dividing the samples into two groups:
> group <- factor(c("1", "1", "1", "1", "1", "1", "2", "2", "2", "2", "2", "2"))
> y$samples$group <- group
# TMM normalization of library sizes
> y <- normLibSizes(y)
> y$samples
> plotMDS(y)
Matrix design
# Define the factors:
> Furin <- factor(c('F', 'F', 'F', 'F', 'F', 'F', 'f', 'f', 'f', 'f', 'f', 'f'))
> Cell <- factor(c('1', '2', '3', '4', '5', '6'))
# Create the data frame
> df <- data.frame(Expression = Furin, Cell = Cell)
> group <- factor(c("1", "1", "1", "1", "1", "1", "2", "2", "2", "2", "2", "2"))
> y$samples$group <- group
> design <- model.matrix (~ 0 + group)
# Estimation of the Negative Binomial dispersions:
> disp <- estimateDisp(y, design, robust=TRUE)
> disp$common.dispersion
> plotBCV(disp)
# Function to apply the model to the data:
> fit <- glmQLFit(y, design, robust=TRUE)
> fit$dispersion
> head(fit$coefficients)
> head(fit$coefficients)
# Calculate QL dispersions
> plotQLDisp(fit)
# Differential gene expression between F vs. f
> levels(group) <- c("group1", "group2")
> contrast <- makeContrasts(group1 - group2, levels =group)
> qlf <- glmQLFTest(fit, contrast=contrast)
> median_logFC <- median(topTags(qlf,n=Inf)$table$logFC)
> print(median_logFC)
> topTags(qlf)
> summary(decideTests(qlf))
> plotMD(qlf)
> abline(h=c(-1, 1), col="blue")
> library(ComplexHeatmap)
> Heatmap(heatmap_data,
```

```
                name = "logCPM",
                show_row_names = TRUE,
                show_column_names = TRUE)
# Gene ontology analysis
>   BiocManager::install("org.Hs.eg.db")
>   library(org.Hs.eg.db)
# Convert gene symbols to Entrez IDs
>   annotated_genes <- mapIds(org.Hs.eg.db, keys = DEGenes,
                              column = "ENTREZID", keytype = "ENSEMBL",
                              multiVals = "first")
# Extract genes with FDR < 0.05
>   DEGenes <- rownames(topTags(qlf, n = Inf)$table[topTags(qlf, n = Inf)$table$FDR <
    0.05, ])
# Check how many DE genes were extracted
>   length(DEGenes)
        [1]     4584
# Filter out any genes that could not be annotated
>   annotated_genes <- annotated_genes[!is.na(annotated_genes)]
# Run GO analysis
>   go <- goana(annotated_genes, species = "Hs")
>   topGO(go, ontology = "BP")
>   topGO(go, ontology = "CC")
>   topGO(go, ontology = "MF")
> print(topGO)
# Gene set testing using the rotation gene set test (ROAST) :
>   library(GO.db)
>   reg_sig.go <- c("GO:0023052")
>   term <- select(GO.db, keys=reg_sig.go, columns="TERM")
            'select()' returned 1:1 mapping between keys and columns
>   term
>   library(AnnotationDbi)
>   Rkeys(org.Hs.egGO2ALLEGS) <- reg_sig.go
# Use org.Hs.egGO2ALLEGS to map GO terms to Entrez Gene IDs
>   mapped_genes <- org.Hs.egGO2ALLEGS[[reg_sig.go]]
>   print(mapped_genes)
>   cont <- makeContrasts(group1 - group2, levels =group)
>   y <- estimateDisp(y, design)
>   plotBCV(y)
>   fr <- fry(y, index=ind, design=design, contrast=cont)
>   fr
>   res <- glmQLFTest(fit, contrast=contrast)
>   barcodeplot(res$table$logFC, ind[[1]], main=names(ind)[1])
>   print(length(ind))
# Retrieve logFC and P-value from glmQLFTest result
>   logFC <- res$table$logFC
>   pvalue <- res$table$PValue
# Define threshold for significance (adjust based on your criteria)
>   threshold <- 0.05
# Extract upregulated genes (positive logFC, p-value < threshold)
>   upregulated <- rownames(res$table)[logFC > 0 & pvalue < threshold]
# Extract downregulated genes (negative logFC, p-value < threshold)
>   downregulated <- rownames(res$table)[logFC < 0 & pvalue < threshold]
# Filter to get genes associated with the GO term "GO:0023052"
>   upregulated_go <- upregulated[upregulated %in% go_genes]
```

```
>   downregulated_go <- downregulated[downregulated %in% go_genes]
# Print or save the results
>   print(upregulated_go)
>   print(downregulated_go)
# Retrieve the total count of genes in the upregulated_go list
>   total_genes <- length(upregulated_go)
 # Print the total count
 >   print(total_genes)      [1]    961
 >   total_genes <- length(downregulated_go)
# Print the total count
>   print(total_genes)       [1]   355
>   library(KEGGREST)
>   print(upregulated_go)
>   kegg_enrichment <- enrichKEGG(gene = upregulated_go, organism = "hsa",
    pvalueCutoff = 0.05)
>   head(kegg_enrichment)
>   kegg_enrichment <- enrichKEGG(gene = downregulated_go, organism = "hsa",
    pvalueCutoff = 0.05)
>   head(kegg_enrichment)
>   surf_recp.go <- c("GO:0007166")
>   surf_recp_genes <- select(org.Hs.eg.db, keys = surf_recp.go, columns = "ENTREZID",
       keytype = "GOALL")
>   head(surf_recp_genes)
>   unique_entrez_ids <- unique(surf_recp_genes$ENTREZID)
      print(unique_entrez_ids)
>   library(clusterProfiler)
>   kegg_enrichment <- enrichKEGG(gene = unique_entrez_ids, organism = "hsa",
    pvalueCutoff = 0.05)
>   head(kegg_enrichment)
>   grw_fct.go <- c("GO:0019838")
>   print(grw_fct.go)
# Check if the corrected GO term is valid
>   grw_fct.go %in% available_go_terms
>   surf_recp_genes <- select(org.Hs.eg.db, keys = grw_fct.go,
       + columns = "ENTREZID",
       + keytype = "GOALL")
>   head(surf_recp_genes)
>   unique_entrez_ids <- unique(surf_recp_genes$ENTREZID)
      print(unique_entrez_ids)
>   library(clusterProfiler)
>   kegg_enrichment <- enrichKEGG(gene = unique_entrez_ids, organism = "hsa",
    pvalueCutoff = 0.05)
>   head(kegg_enrichment)
# Convert KEGG enrichment results to a data frame
>   kegg_enrichment_df <- as.data.frame(kegg_enrichment)
# Print the first 20 rows of the data frame
>   print(head(kegg_enrichment_df, 20))
# Check the structure of the KEGG enrichment result
>   str(kegg_enrichment)
# Convert the KEGG enrichment result to a data frame
>   kegg_enrichment_df <- as.data.frame(kegg_enrichment)
# Extract the row corresponding to the "Human papillomavirus infection" pathway (ID:
    hsa05165)
>   hpv_row <- kegg_enrichment_df[kegg_enrichment_df$ID == "hsa05165", ]
```

```
# Extract the gene IDs (usually stored in the `geneID` column)
>   hpv_genes <- hpv_row$geneID
# The gene IDs are usually stored as a single string, separated by "/"
>   hpv_genes_list <- unlist(strsplit(hpv_genes, "/"))
# Print the list of gene IDs involved in the HPV pathway
>   print(hpv_genes_list)
# Convert Entrez IDs to gene symbols
>   library(org.Hs.eg.db)
>   hpv_gene_symbols <- bitr(hpv_genes_list, fromType = "ENTREZID", toType = "SYM-
    BOL", OrgDb = org.Hs.eg.db)
# View the gene symbols
>   print(hpv_gene_symbols)
# Save the genes involved in the HPV pathway to a CSV file
>   write.csv(hpv_gene_symbols, file = "hpv_pathway_genes.csv", row.names = FALSE)
# File with the list of the 64 genes as entrezgene_id = hpv_genes_list
>   kegg_pathway_enrichment <- enrichKEGG(gene = hpv_genes_list, organism = "hsa",
    pvalueCutoff = 0.05)
>   head(kegg_pathway_enrichment)
# Load the ReactomePA package
>   library(ReactomePA)
# Perform Reactome enrichment analysis using the Entrez IDs
>   reactome_enrichment <- enrichPathway(gene = hpv_genes_list,
                                          organism = "human",
                                          pvalueCutoff = 0.05)
# View the top Reactome pathways
>   head(reactome_enrichment)
Violin plots:
> library(ggplot2)
> library(tidyr)
> violin_plot_long <- violin_plot %>% pivot_longer(cols = -Tissue, names_to = "Gene", val-
ues_to = "nTPM")
> summary_stats <- violin_plot_long %>%
   group_by(Gene) %>%
   summarise(mean = mean(nTPM), sd = sd(nTPM)) %>%
   mutate(ymin = mean - sd, ymax = mean + sd)
> ggplot(violin_plot_long, aes(x = Gene, y = nTPM, fill = Gene)) +
   geom_violin(trim = FALSE, alpha = 0.7) +   # Violin plot
   geom_jitter(aes(color = Gene), size = 2, alpha = 0.8, width = 0.2) +   # Jitter points
   geom_point(data = summary_stats, aes(x = Gene, y = mean),
              inherit.aes = FALSE, color = "black", size = 3, shape = 18) +   # Add mean
points
   geom_errorbar(data = summary_stats, aes(x = Gene, ymin = ymin, ymax = ymax),
                 inherit.aes = FALSE, width = 0.2, color = "black") +   # Add error bars
   labs(title = "nTPM Distribution Across Genes with Mean and SD",
        x = "Gene", y = "nTPM") +
   theme_minimal() +
   theme(
     axis.text.x = element_text(size = 12),
axis.title = element_text(size = 14),
legend.position = "none" )
```
